# Clinical Outcomes and Factors Associated with MACCE in Chinese Patients Undergoing Single-Hospitalization PCI and Transfemoral TAVR

**DOI:** 10.3390/jcdd13080394

**Published:** 2026-08-17

**Authors:** Tong Tan, Alimujiang Awakeri, Hao Cui, Enjun Zhu, Yongqiang Lai

**Affiliations:** Beijing Anzhen Hospital, Capital Medical University, Beijing Institute of Heart, Lung and Blood Vascular Diseases, Beijing 100029, China

**Keywords:** percutaneous coronary intervention, transcatheter aortic valve replacement, aortic stenosis, coronary artery disease, major adverse cardiovascular and cerebrovascular events

## Abstract

Background: The incidence and predictors of major adverse cardiovascular and cerebrovascular events (MACCE) in patients undergoing percutaneous coronary intervention (PCI) and transcatheter aortic valve replacement (TAVR) during the same hospitalization remain poorly characterized, particularly in Asian populations. Methods: This single-center retrospective study included 164 consecutive patients who underwent PCI and transfemoral TAVR during the same hospitalization at Beijing Anzhen Hospital between June 2018 and October 2023. The primary objective was to evaluate the incidence of MACCE, and logistic regression analyses were used to identify its predictors. Results: Among 164 patients, the mean age was 73.9 ± 7.2 years, and 57.9% were male. Device success was 97.6% and procedural success was 89.6%. MACCE occurred in 28 patients (17.1%) within the index hospitalization or 30 days after discharge. Independent predictors of MACCE were lower baseline left ventricular ejection fraction (LVEF; OR = 0.955, 95% CI 0.920–0.992; *p* = 0.017) and mixed aortic stenosis and regurgitation (AS + AR; OR = 6.360, 95% CI 1.035–38.993; *p* = 0.038). Conclusions: A single-hospitalization PCI and TAVR strategy appeared feasible and was associated with acceptable short-term clinical outcomes in carefully selected Chinese candidates, with lower baseline LVEF and mixed AS and AR as independent predictors of MACCE. The observed clinical outcomes should be interpreted descriptively because of the lack of a contemporaneous comparator group; large-scale prospective studies are needed to further evaluate the safety and clinical benefit of this “one-stop” paradigm.

## 1. Introduction

With the rapid evolution of transcatheter valve therapies, transcatheter aortic valve replacement (TAVR) has become an established treatment option for patients with severe aortic stenosis (AS), and current international guidelines have further expanded its indications. According to the 2025 ESC/EACTS Guidelines for the Management of Valvular Heart Disease, TAVR has a Class I recommendation for patients aged ≥70 years with severe tricuspid aortic stenosis who are anatomically suitable [1]. Aortic valve disease (AVD) frequently coexists with coronary artery disease (CAD), and more than half of patients undergoing TAVR have significant coronary lesions that may require percutaneous coronary intervention (PCI) [2], which continues to complicate treatment decision-making. Current guidelines recommend concomitant surgical aortic valve replacement and coronary artery bypass grafting in patients with a primary indication for valve surgery and coronary artery stenosis ≥70%, whereas for patients undergoing transcatheter valve intervention, PCI may be considered for significant proximal coronary stenosis (≥70%) based on individualized Heart Team assessment [1]. In selected patients, a fully percutaneous treatment strategy combining PCI and TAVR may be particularly relevant for elderly individuals, those at high or prohibitive surgical risk, and those with anatomy favorable for transfemoral access, in whom avoidance of sternotomy, cardiopulmonary bypass, and prolonged postoperative recovery may be beneficial. Although the optimal timing of PCI (before/during/after TAVR) remains uncertain, transfemoral TAVR combined with PCI during the same hospitalization represents a pragmatic one-stop strategy, which may reduce the need for repeated admissions, multiple vascular access procedures, and delayed treatment, while offering a less invasive alternative to conventional SAVR with CABG. Previous studies have suggested that this one-stop strategy is technically feasible and can be performed with acceptable short-term outcomes in selected intermediate- and high-risk patients [3]. However, these data are derived almost exclusively from Western populations, and evidence from Asian cohorts remains scarce. Moreover, few studies have specifically examined major adverse cardiovascular and cerebrovascular events (MACCE) and their predictors in patients undergoing PCI with TAVR [4]. To address these gaps, the present study aimed to evaluate the incidence of early MACCE and to identify baseline or pre-procedural factors associated with it in Chinese patients undergoing PCI and transfemoral TAVR during the same hospitalization. This study provides additional evidence to facilitate evidence-based decision-making in the management of coexisting coronary and valvular heart disease.

## 2. Materials and Methods

### 2.1. Study Design and Patient Population

This was a single-center, retrospective observational study conducted at Beijing Anzhen Hospital. Between June 2018 and October 2023, consecutive patients who underwent PCI and TAVR during the same hospitalization were included in the analysis. Patients were identified from the institutional TAVR registry and electronic medical records. Data collection included patient demographics, comorbidities, echocardiographic findings, procedural details, and clinical outcomes.

The inclusion criteria were defined as follows: (1) patients aged ≥18 years; (2) a diagnosis of severe AS, aortic regurgitation (AR), or mixed AS/AR requiring TAVR, together with concomitant obstructive CAD requiring revascularization based on Heart Team consensus. Obstructive CAD was defined as ≥70% diameter stenosis in a major epicardial coronary artery or ≥50% stenosis in the left main stem, primarily based on visual angiographic estimation during invasive coronary angiography. Physiological assessment using fractional flow reserve or instantaneous wave-free ratio was performed selectively at the operator’s discretion but was not routinely required; (3) performance of PCI and transfemoral TAVR within a single hospital admission; (4) PCI performed before or concomitantly with TAVR during the same hospitalization, based on the Heart Team decision. Exclusion criteria were: (1) prior surgical or transcatheter aortic valve intervention; (2) other concomitant cardiac surgeries; and (3) incomplete procedural or outcome data.

Follow-up was conducted at 30 days, 3 months after discharge, and at subsequent annual visits or telephone interviews, when available. Thirty-day outcomes, including all components of MACCE, were ascertained for the entire cohort through review of institutional electronic medical records, hospital readmission databases, and emergency department records, and were therefore not affected by loss to follow-up. Patients without post-discharge data beyond 30 days were considered lost to follow-up. All available follow-up echocardiographic parameters and clinical events were cross-checked against the institutional medical records to ensure accuracy.

### 2.2. Procedural Technique

**Figure 1:**
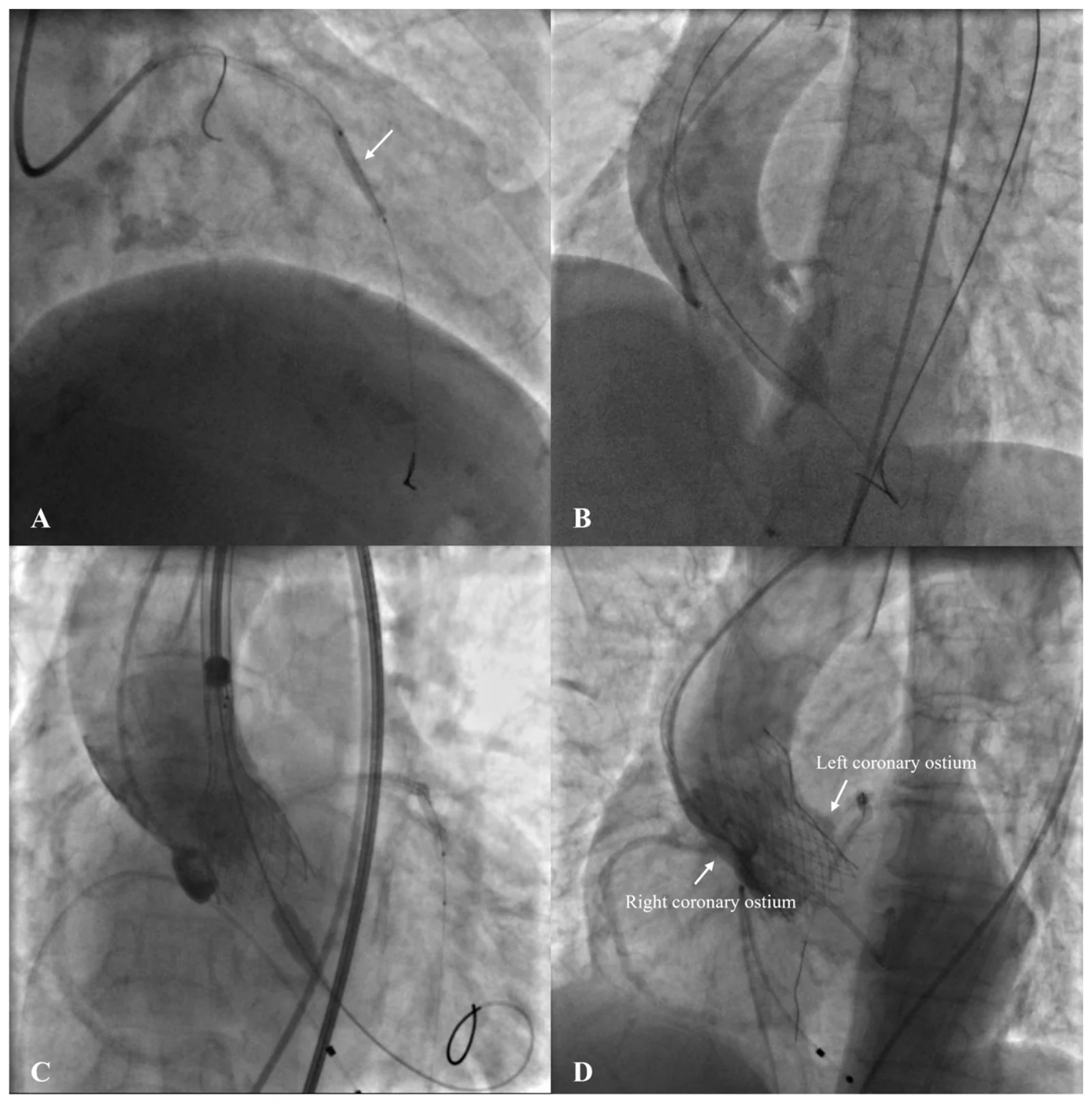
Representative procedural images from a patient undergoing concomitant PCI and TAVR. (**A**) Percutaneous coronary intervention of an obstructive coronary lesion (arrow). (**B**) Retrograde crossing of the stenotic aortic valve with a dedicated guidewire. (**C**) Fluoroscopic positioning of the transcatheter heart valve across the aortic annulus; note the simultaneous coronary angiography performed to verify ostial patency and prevent coronary obstruction before final release. (**D**) Final fluoroscopic image after transcatheter heart valve implantation. The arrows indicate the left and right coronary ostia, demonstrating preserved coronary patency following valve deployment.

All treatment strategies were determined by a multidisciplinary Heart Team considering operative risk, valve pathology, coronary anatomy, and patient preference. The procedures were performed as previously reported [5]. Coronary angiography was routinely performed as a separate diagnostic procedure prior to TAVR in all patients. In cases where significant coronary artery disease was identified, the procedure was not routinely continued with immediate PCI. Instead, transthoracic echocardiography and coronary findings were reviewed by the multidisciplinary Heart Team to determine the optimal revascularization strategy, taking into account the potential suitability for surgical revascularization with concomitant aortic valve replacement. Once a single-hospitalization PCI–TAVR strategy had been agreed upon by the Heart Team and the patient, all procedures were performed in a hybrid catheterization laboratory. In this study cohort, a “PCI-first” strategy was consistently utilized, with coronary revascularization performed prior to TAVR during the same hospitalization. This approach included two coronary revascularization strategies: staged PCI followed by TAVR and concomitant PCI performed during the same procedural session as TAVR. The rationale for this strategy was primarily based on procedural risk reduction. When coronary lesions were considered clinically relevant, particularly significant proximal or major epicardial stenosis, untreated CAD was judged to potentially increase the risk of peri-procedural ischemia or hemodynamic instability during valve crossing, balloon valvuloplasty, rapid ventricular pacing, and valve deployment. In addition, coronary access after TAVR may be more challenging depending on coronary height, sinus anatomy, commissural alignment, and valve frame design. The choice of strategy was determined by the Heart Team. In general, concomitant PCI was considered in patients with clinically significant and technically straightforward lesions requiring immediate revascularization, whereas a staged approach was preferred in patients with complex coronary anatomy, higher anticipated contrast load, impaired renal function, or increased bleeding risk. The final decision was individualized based on coronary lesion complexity, hemodynamic status, renal function, and overall procedural risk. In patients undergoing a staged PCI strategy, coronary revascularization was performed on a separate day prior to TAVR. In selected patients, PCI and TAVR were performed during the same procedural session, typically in the setting of hemodynamic instability or when immediate coronary revascularization was deemed necessary. During PCI, obstructive coronary lesions were addressed using standard percutaneous techniques. Revascularization primarily involved balloon angioplasty and drug-eluting stent deployment (Figure 1A). Subsequently, TAVR was performed. In both staged and concomitant strategies, the risk of coronary obstruction was systematically assessed before TAVR using pre-procedural computed tomography, including coronary ostial height, sinus of Valsalva dimensions, sinotubular junction anatomy, leaflet calcification, and the spatial relationship between previously implanted coronary stents and the anticipated transcatheter valve frame. For patients considered at increased risk of coronary compromise, protective strategies, such as pre-emptive coronary guidewire placement and preparation of an undeployed coronary stent for potential chimney stenting, were planned on an individualized basis. After navigating the native aortic valve with a dedicated guidewire, the bioprosthetic valve was advanced across the aortic annulus (Figure 1B). Coronary patency was reassessed after valve positioning, particularly in patients with low coronary ostial height or extensive leaflet calcification. Once optimal positioning and hemodynamic stability were confirmed, the transcatheter heart valve was fully deployed (Figure 1C,D). Coronary ostial patency was routinely assessed by angiography immediately after valve deployment. In patients with hemodynamic instability, valve deployment was prioritized to rapidly restore forward flow, with procedural modifications aimed at minimizing rapid ventricular pacing duration, limiting repeated repositioning, and avoiding prolonged intraprocedural manipulation. Final procedural success was confirmed by angiography and echocardiography.

### 2.3. Endpoint Definition

The primary endpoint was MACCE, defined as the occurrence of at least one of the following events during the index hospitalization or within 30 days after discharge: all-cause death, myocardial infarction, stroke, rehospitalization for heart failure, repeat revascularization, cardiogenic shock, major bleeding, or cardiac arrest. MACCE was assessed at the patient level; patients experiencing multiple component events were counted only once in the composite endpoint. Individual MACCE components were not mutually exclusive and were reported separately. Secondary endpoints focused on procedural performance and device-related outcomes, as recommended by VARC-3. Device success required successful valve deployment with appropriate positioning and function, absence of device-related complications, and no need for unplanned additional valve implantation or surgical intervention. Procedural success was also defined as successful completion of the planned TAVR procedure without intraprocedural death or major procedure-related complications. Given that all patients also underwent PCI, procedural success in our study additionally required successful coronary revascularization for target lesions, defined as restoration of adequate coronary flow with TIMI grade 3 flow and the absence of major periprocedural PCI-related complications.

### 2.4. Statistical Analysis

All statistical analyses were performed using R version 4.5.1 (R Foundation for Statistical Computing, Vienna, Austria) in R software (RStudio version 2025.09.1). Continuous variables were expressed as mean ± standard deviation or median with interquartile range (IQR), depending on the data distribution, assessed by the Shapiro–Wilk test. Categorical variables were presented as counts and percentages. Comparisons between groups were performed using the *t*-test or Mann–Whitney U test for continuous variables, and the chi-square or Fisher’s exact test for categorical variables, as appropriate. The principal objective of this study was to evaluate the incidence of MACCE and baseline or pre-procedural factors associated with MACCE. The primary endpoint, MACCE, was analyzed as a binary outcome variable. Univariable logistic regression analyses were initially performed. Given the limited number of MACCE, the multivariable model was also restricted to variables selected from univariable analysis to avoid overfitting. All variables with *p* < 0.05 in univariable analysis were entered into the multivariable model, with one variable retained from each set of collinear variables to avoid multicollinearity. Post-procedural complications, including acute kidney injury (AKI), were not considered candidate variables for this model. The events-per-variable (EPV) ratio was calculated to assess the potential risk of overfitting. A sensitivity analysis was performed using Firth’s penalized likelihood logistic regression to account for small sample bias and rare outcome events. Statistical significance was defined as a two-tailed *p*-value < 0.05.

## 3. Results

### 3.1. Baseline Characteristics of the Overall Cohort

A total of 164 patients who underwent single-hospitalization PCI and TAVR were included. Detailed characteristics of the overall cohort are presented in Table 1. The mean age was 73.9 ± 7.2 years, and 95 patients (57.9%) were male. At presentation, 112 patients (68.3%) were in New York Heart Association (NYHA) functional class III or IV, and the median EuroSCORE II was 4.8% (IQR, 3.3–8.5). The cohort was characterized by a high burden of comorbidities. Hypertension was present in 114 patients (69.5%), diabetes mellitus in 52 (31.7%), and chronic kidney disease in 37 (22.6%). Significant left main (LM) coronary artery stenosis (>50%), left anterior descending artery (LAD) stenosis (>70%), and isolated proximal left circumflex artery (LCX) and/or right coronary artery (RCA) stenosis (>70%) were observed in 16 (9.8%), 88 (53.7%), and 73 (44.5%) patients, respectively. Previous cardiac intervention was present in 28 patients (17.1%). Among these 28 patients, 25 had undergone previous coronary revascularization via percutaneous approaches (89.3% of patients with previous cardiac intervention), and 1 patient each had a history of coronary artery bypass grafting, mitral valve replacement, and radiofrequency ablation (3.6% each).

On preoperative echocardiographic assessment, severe AS was present in 157 patients (95.7%), while 13 patients (7.9%) had severe AR. Six patients (3.7%) had combined severe aortic stenosis and regurgitation. All patients were evaluated by a multidisciplinary Heart Team and deemed appropriate candidates for TAVR, with patients with AR treated using self-expanding devices with dedicated anchoring mechanisms (e.g., J-Valve) in accordance with available evidence for this indication. Severe mitral regurgitation was observed in 24 patients (14.6%). Concomitant mitral intervention was not pursued in these patients, given the expectation that functional MR may improve following relief of the primary hemodynamic burden through TAVR and coronary revascularization. The median left ventricular ejection fraction (LVEF) was 61.0 (54.0, 65.0)%, and the median peak aortic jet velocity was 452.5 (412.0, 501.2) cm/s. Baseline demographic characteristics, major comorbidities, coronary lesion distribution, renal function, and preoperative echocardiographic parameters were comparable between patients who underwent PCI and TAVR on the same day and those who underwent the two procedures on different days during the same hospitalization, with no statistically significant differences observed.

### 3.2. Procedural Characteristics and Outcomes

Thirty-four patients (20.7%) underwent PCI and TAVR on separate dates during the same hospitalization, while the remaining patients (79.3%) received both procedures during a single procedural course. All PCI procedures were successfully performed, with effective treatment of the target lesions. The median numbers of coronary lesions and implanted stents per patient were 2.0 (1.0, 3.0) and 1.0 (1.0, 2.0), respectively. The median number of balloons used for angioplasty per procedure was 2.0 (1.0, 3.0). Drug-coated balloons were used in 3 patients (1.8%) as an alternative revascularization strategy. The SYNTAX score decreased significantly after PCI, from 11.0 (5.0, 18.8) at baseline to 0.0 (0.0, 2.0) post-procedure (*p* < 0.01). Complete anatomical revascularization, defined as a residual SYNTAX score of 0, was achieved in 112 patients (68.3%). No PCI-related procedural complications were observed. During the TAVR procedures, 1 patient (0.6%) experienced compromised left coronary flow during valve positioning, and valve deployment was therefore aborted. After consultation with the patient’s family, no conversion to surgical aortic valve replacement or further transcatheter intervention was performed, and the patient was managed conservatively with optimized medical therapy. Among the remaining patients, single valve implantation was successful, without the need for a second prosthesis (valve-in-valve) in any case. Implanted prostheses included the SAPIEN series (Edwards Lifesciences, Irvine, CA, USA; *n* = 4), the Evolut series (Medtronic, Minneapolis, MN, USA; *n* = 5), the Taurus series (Peijia Medical Technology Co., Ltd., Suzhou, China; *n* = 31), VenusA-Valve (Venus Medtech Inc., Hangzhou, China; *n* = 96), VitaFlow (Shanghai MicroPort CardioFlow Medtech Co., Ltd., Shanghai, China; *n* = 24), Renatus (Beijing Balance Medical Technology Co., Ltd., Beijing, China; *n* = 2), and J-Valve (Suzhou Jiecheng Medical Technology Co., Ltd., Suzhou, China; *n* = 1). One patient (0.6%) developed moderate paravalvular leak (PVL) immediately after valve implantation and subsequently underwent successful PVL closure, while two additional patients (1.2%) were found to have moderate PVL prior to discharge and were managed conservatively with medical therapy. There were no intraprocedural deaths or conversions to open surgery. Device-related complications, such as coronary obstruction, were all negative, resulting in a device success rate of 97.6% (160/164) according to VARC-3 definitions.

With respect to in-hospital outcomes, AKI occurred in 18 patients (11.0%), of whom 4 required continuous renal replacement therapy. The incidence of AKI was similar between patients undergoing concomitant PCI and TAVR and those undergoing staged PCI before TAVR during the same hospitalization (14/130 [10.8%] vs. 4/34 [11.8%], *p* = 1.000). Mechanical circulatory support, including intra-aortic balloon pump (IABP) and extracorporeal membrane oxygenation (ECMO), was required in 4 patients (2.4%, IABP in 1 case and ECMO in 4 cases). Among them, two patients presented with severely impaired left ventricular systolic function (LVEF < 40%) pre-procedurally and developed persistent low cardiac output following TAVR; one patient was supported with combined IABP and ECMO, and the other with ECMO alone. One additional patient with pre-existing moderate-to-severe mitral regurgitation and interventricular septal hypertrophy but without evidence of hypertrophic obstructive cardiomyopathy or systolic anterior motion received prophylactic ECMO to prevent anticipated hemodynamic instability after valve implantation. The remaining patient developed acute neurological complications and complete atrioventricular block post-procedure, accompanied by refractory hemodynamic instability, and was managed with rescue ECMO. Other adverse events comprised myocardial infarction in 1 patient (0.6%), blood transfusion in 19 patients (11.6%), deep vein thrombosis in 2 patients (1.2%), gastrointestinal bleeding in 4 patients (2.4%), stroke in 6 patients (3.7%), and sepsis in 2 patients (1.2%). Permanent pacemaker implantation (PPI) was required in 2 patients (1.2%) due to high-grade conduction disturbances, and 6 patients (3.7%) died during the index hospitalization. Overall, procedural success was achieved in 89.6% of patients (147/164). At discharge, the median LVEF was 60.0% (54.0, 65.0), which was not significantly different from baseline (*p* = 0.380). In contrast, the peak aortic jet velocity was significantly reduced to 229.0 (186.8, 264.8) cm/s compared with pre-procedural values (*p* < 0.001). Detailed procedural characteristics, early clinical outcomes, and the individual components of MACCE are summarized in Table 2.

Thirty-day outcomes were ascertained for all patients through institutional medical records. All surviving patients were subsequently scheduled for longer-term follow-up, during which 16 patients were lost to follow-up beyond the 30-day window. During a median follow-up of 12.2 (6.3, 29.5) months, LVEF significantly improved to 64.0% (60.0, 67.0) compared with baseline (*p* < 0.001). In parallel, significant reverse remodeling was observed, with reductions in left ventricular end-diastolic diameter to 47.0 (44.0, 50.0) mm and left ventricular end-systolic diameter to 29.5 (28.0, 32.0) mm (both *p* < 0.05). Among the 24 patients with severe MR at baseline, only two had persistent severe MR at follow-up, but both experienced marked symptomatic improvement. The remaining patients showed improvement in MR severity and clinical symptoms after TAVR. New-onset moderate PVL occurred in 3 patients, and 3 patients required PPI during follow-up. Additionally, 12 patients experienced rehospitalization for heart failure, and 4 patients died during the follow-up period.

### 3.3. Factors Associated with MACCE

Overall, 28 patients (17.1%) experienced at least one MACCE during the index hospitalization or within 30 days after discharge. The incidence of MACCE was numerically higher when PCI and TAVR were performed on the same day than on different days within the same hospitalization (19.2% vs. 8.8%; unadjusted OR 2.46, 95% CI 0.70–8.67; *p* = 0.203). Patients in the MACCE group had a higher EuroSCORE II than those without MACCE (6.6% [4.7%, 11.7%] vs. 4.4% [3.0%, 8.2%], *p* = 0.009), a higher prevalence of severe AR (17.9% vs. 5.9%, *p* = 0.049), and worse baseline cardiac function, as reflected by a higher proportion of patients with NYHA ≥ III and a lower LVEF (55.0% [46.0, 58.0] vs. 61.0% [55.2, 66.0], *p* < 0.001). Regarding post-procedural complications, the incidence of AKI was significantly higher in patients who developed MACCE than in those who did not (32.1% [9/28] vs. 6.6% [9/136], *p* < 0.001). In univariable analyses, valve frame length was not significantly associated with peri-procedural MACCE. Patients receiving short-frame valves showed numerically lower odds of MACCE compared with those receiving long-frame valves (OR 0.80, 95% CI 0.04–4.96, *p* = 0.842), although this difference did not reach statistical significance. Similarly, baseline and residual SYNTAX score and complete anatomical revascularization were not significantly associated with MACCE. However, lower pre-procedural LVEF and the presence of severe combined AS and AR were significantly associated with MACCE. Among the other baseline variables, EuroSCORE II and NYHA functional class met the univariable screening criterion of *p* < 0.05. Although EuroSCORE II and NYHA functional class met the univariable screening criterion, they were not entered into the final model because EuroSCORE II explicitly incorporates both NYHA functional class and LVEF, resulting in overlapping clinical information among these measures. Baseline LVEF was retained as the objective continuous measure of ventricular systolic function and showed the strongest univariable association with MACCE among these measures. Mixed severe AS with AR was retained because it represented a distinct valve-disease phenotype. Therefore, given the limited number of MACCE and the potential collinearity among candidate predictors, the final multivariable model was restricted to baseline LVEF and mixed severe AS with AR. Following adjustment for relevant covariates in the multivariable logistic regression model, these factors remained independently associated with the primary outcome (Table 3). The final model contained two parameters and 28 MACCE, corresponding to an EPV of 14.0. The estimates were derived from the same multivariable model. Specifically, each 1% increase in baseline LVEF was associated with a decreased risk of MACCE (OR 0.955; 95% CI, 0.920–0.992; *p* = 0.017), implying that lower pre-procedural LVEF served as an independent predictor of adverse events. Furthermore, severe concomitant AS and AR conferred a significantly higher risk of MACCE than those without mixed valvular disease (OR 6.363; 95% CI, 1.112–36.405; *p* = 0.038). Note that the wide confidence interval reflects the limited number of patients with this valve phenotype. Sensitivity analyses using Firth’s penalized likelihood logistic regression yielded consistent results, confirming the robustness of the main findings. Lower pre-procedural LVEF remained associated with increased MACCE risk (OR 0.957, 95% CI 0.922–0.992; *p* = 0.018), while mixed severe AS with AR (OR 6.106, 95% CI 1.133–32.740; *p* = 0.036) remained an independent predictor of MACCE.

## 4. Discussion

AVD and CAD represent the leading cardiovascular morbidities in the aging population. Epidemiological data from a large elderly cohort (e.g., the DANCAVAS study) indicate that approximately 90% of individuals with aortic valve calcification also exhibit concomitant coronary artery calcification, underscoring the shared calcific pathophysiology between these two conditions, characterized by endothelial dysfunction, lipid infiltration, chronic inflammation, and progressive calcification [6,7]. They also share some risk factors such as hypertension and dyslipidemia. This clinical intersection carries profound prognostic weight, as the coexistence of AVD and CAD is associated with significantly higher cardiovascular morbidity and mortality than either condition in isolation [8]. Historically, the gold standard for managing patients with this dual pathology was combined surgical aortic valve replacement and coronary artery bypass grafting. With the rapid evolution of transcatheter technologies and the shift toward minimally invasive paradigms, the combination of TAVR and PCI has emerged as a therapeutic alternative for selected older or intermediate-risk patients with concomitant AVD and CAD, particularly when anatomical suitability, comorbidities, patient preference, and Heart Team assessment favor a less invasive strategy. In the present cohort, the indication for TAVR was primarily symptom-driven following multidisciplinary Heart Team assessment, rather than based on anatomical suitability alone. Accordingly, the relatively high proportion of patients presenting with NYHA functional class III/IV likely reflects the real-world referral pattern of patients with advanced symptomatic disease requiring concomitant coronary revascularization [9], which should be considered when extrapolating our findings to less symptomatic TAVR populations. Nevertheless, the role and extent of coronary revascularization in this setting remain a matter of ongoing debate. Current consensus guidelines restrict the recommendation for PCI in the TAVR population to those with obstructive lesions (stenosis >70%) localized in proximal coronary segments [1,10]. In our cohort, complete anatomical revascularization was achieved in 68.3% of patients. Incomplete revascularization was mainly related to diffuse distal disease, small vessels, severe calcification, chronic total occlusions, or a Heart Team decision to prioritize clinically relevant proximal lesions while minimizing contrast exposure and procedural risk. Previous studies have suggested that residual coronary disease, especially a higher residual SYNTAX score, may be associated with worse outcomes after TAVR or complex PCI [11]. However, in our analysis, complete anatomical revascularization was not significantly associated with MACCE. This may reflect the short follow-up period, limited number of events, and individualized PCI strategy focused on clinically important lesions rather than mandatory complete anatomical revascularization.

The optimal timing for coronary revascularization in patients undergoing TAVR remains controversial. A national analysis using the Nationwide Readmissions Database reported that concomitant PCI/TAVR was associated with similar in-hospital mortality compared with staged procedures, along with reduced total hospital stay and cost [12]. Furthermore, meta-analyses have consistently shown no significant differences in stroke, myocardial infarction, or bleeding between concomitant and staged PCI/TAVR strategies, suggesting comparable procedural safety [13,14]. However, recent evidence suggests that this neutrality may not apply to all patient subsets. For instance, the ASCoP registry [15] indicated that for complex or high-risk coronary interventions, a concomitant approach may exacerbate peri-procedural risks and composite adverse outcomes. Rheude et al. reported that performing PCI and TAVR during the same session was associated with higher mortality [16]. Another concern with the concomitant approach is the potential for AKI, likely driven by the cumulative contrast media volume and prolonged procedural duration within a single session [17,18]. Given that AKI after TAVR is a common and prognostically important complication and is routinely assessed as a short-term safety outcome in TAVR cohorts, careful attention to renal injury is warranted when considering concomitant procedures [19,20]. Our study provides descriptive real-world data on the feasibility and short-term outcomes of performing PCI and TAVR within a single hospitalization in a Chinese population. The overall procedural success rate was high, with a low incidence of in-hospital mortality and complications such as stroke, myocardial infarction, and life-threatening bleeding, comparable to previous reports [21,22,23]. Notably, in the Nationwide Readmissions Database analysis by Ghrair et al. [22], combined TAVR and PCI was associated with higher in-hospital mortality than isolated TAVR (4.5% vs. 1.7%), as well as higher rates of cardiogenic shock (9.4% vs. 2.1%), mechanical circulatory support use (6.8% vs. 0.7%), acute kidney injury (25.5% vs. 11.5%), and bleeding (25.2% vs. 18.1%). In another national analysis, Tran et al. [12] reported that concomitant PCI/TAVR had similar risk-adjusted in-hospital mortality compared with staged PCI/TAVR, while staged PCI/TAVR was associated with higher odds of acute kidney injury. Taken together, these findings suggest that the early mortality and need for mechanical circulatory support observed in our cohort should be interpreted in the context of patient complexity and case mix rather than attributed solely to the single-hospitalization strategy. At follow-up, patients demonstrated significant improvement in LVEF and favorable reverse remodeling, consistent with the hemodynamic and functional benefit of this single-hospitalization PCI–TAVR strategy; however, these echocardiographic changes should be interpreted as descriptive and hypothesis-generating rather than definitive evidence of long-term benefit. Our findings suggest that performing PCI and TAVR within a single hospital admission appears feasible and is associated with acceptable short-term outcomes in appropriately selected patients with severe AVD and concomitant CAD. This approach may reduce repeated vascular access, shorten hospital stay, and optimize resource utilization. Within our cohort, MACCE rates were numerically higher when PCI and TAVR were performed on the same day than on different days within the same hospitalization (19.2% vs. 8.8%, *p* = 0.203). However, this difference did not reach statistical significance and should not be interpreted as evidence of a clinically meaningful difference between strategies. Given the limited sample size, the lack of statistical significance may reflect insufficient power and a potential type II error rather than true equivalence. Concomitant TAVR and PCI may impose greater procedural burden and overlapping peri-procedural risks, whereas a staged approach may allow clinical stabilization and reassessment. Therefore, these findings should be considered hypothesis-generating only. Larger, adequately powered studies are needed to determine whether procedural timing influences clinical outcomes.

Patient selection should integrate anatomical complexity, renal reserve, and hemodynamic stability. Patients with less complex coronary anatomy (single-vessel or ostial lesions), preserved renal function, and tolerance for a single contrast load are ideal candidates for a concomitant strategy. Perez et al. further emphasized that unstable patients or those at high risk for coronary occlusion (e.g., due to a low coronary ostial height) may also benefit from simultaneous PCI and TAVR [24]. Even among patients with left main disease, the TAVR-LM registry reported comparable one-year mortality and stroke rates between TAVR plus left main PCI and isolated TAVR [25]. From a procedural perspective, performing PCI prior to or concurrently with TAVR is generally favored over post-TAVR revascularization. Inadequate revascularization beforehand may compromise myocardial perfusion during valve deployment, thereby predisposing patients to intra-procedural hemodynamic collapse. Moreover, the presence of long-frame transcatheter valves may limit future coronary access [26]. Clinical evidence supports this proactive approach, as a pre-TAVR PCI strategy targeted at achieving a residual SYNTAX score of <8 has been demonstrated to improve one-year survival and significantly reduce the incidence of MACCE [11]. Additionally, concomitant PCI with TAVR may serve as a rescue strategy for hemodynamically unstable patients. In cases where severe AS and critical CAD coexist and precipitate cardiogenic shock, a staged approach may be infeasible due to the inability to tolerate a delay between interventions. In such scenarios, simultaneous PCI and TAVR have been shown to restore hemodynamic stability and improve short-term survival [5,27]. Future research should aim to identify early markers of hemodynamic decompensation and delineate which high-risk subgroups derive the greatest benefit from a concomitant strategy.

Beyond procedural feasibility, our multivariable analysis identified two independent predictors of MACCE following PCI and TAVR performed during the same hospitalization—lower baseline LVEF and severe combined AS and AR. Reduced baseline LVEF has consistently been associated with adverse outcomes after cardiovascular interventions. Such patients exhibit diminished myocardial reserve and are more vulnerable to hemodynamic compromise during valve deployment and to the additional ischemic and contrast load imposed by PCI, predisposing them to early cardiovascular events [28,29]. Pre-procedural optimization of ventricular function should be considered to improve peri-procedural safety, particularly when achieved through intensified medical therapy, hemodynamic stabilization, or staged balloon aortic valvuloplasty for patients with markedly reduced LVEF. In addition, early use of mechanical circulatory support in high-risk candidates has been described in the context of emergency TAVR for cardiogenic shock [30], and its role in elective concomitant PCI and TAVR merits prospective evaluation. Finally, severe concomitant AS and AR emerged as an independent predictor of MACCE, reflecting the unique pathophysiology of mixed aortic valve disease (MAVD). This “dual-load” state, in which pressure and volume overload coexist, subjects the left ventricle to divergent and potentially maladaptive remodeling stimuli. Compared with pure AS, in which the LV is primarily adapted to chronic pressure overload through concentric hypertrophy, MAVD adds a regurgitant volume burden to an already hypertrophied and less compliant ventricle. As a result, even a modest AR component may disproportionately increase LV end-diastolic volume and pressure, left atrial pressure, and pulmonary congestion, while simultaneously reducing effective forward stroke volume. This makes the MAVD ventricle less tolerant of peri-procedural preload–afterload shifts than the ventricle with isolated AS [31]. The exaggerated hypertrophy and progressive myocardial fibrosis associated with MAVD impair both diastolic compliance and contractile reserve [32]. Furthermore, the abrupt hemodynamic transition following TAVR may provoke acute maladaptive responses. In MAVD, the chronically enlarged LV is adapted to high end-diastolic volumes. The sudden elimination of regurgitation and afterload reduction post-TAVR can trigger a rapid “afterload–preload inversion.” This instability can precipitate transient systolic dysfunction or ventricular arrhythmias during the early post-procedural phase, contributing to heightened peri-procedural risk even after technically successful implantation [33,34]. Consistent with these mechanisms, prior studies have shown that MAVD is associated with advanced ventricular remodeling and a higher burden of adverse clinical events, supporting MAVD as a distinct high-risk phenotype rather than a simple combination of AS and AR [32,35,36].

This study has several limitations that should be acknowledged. First, it was a single-center, retrospective analysis, which may have introduced selection bias and limited the generalizability of the findings to other populations or institutions. Second, although this study represents the largest Chinese cohort to date of patients undergoing PCI and TAVR during the same hospitalization, the overall sample size remains relatively small, and the number of events was limited, potentially reducing the statistical power of multivariable analyses. In addition, most transcatheter heart valves used in this cohort were domestic valve systems, including VenusA, VitaFlow, and Taurus, whereas Edwards Sapien and Medtronic Evolut valves were used in only a small proportion of patients. Therefore, the present findings may not be directly generalizable to centers or regions where Sapien or Evolut systems are predominantly used, particularly given potential differences in valve frame design, commissural alignment, coronary access, and procedural strategy. Third, longer-term follow-up was incomplete and was not systematically performed according to a uniform protocol. Follow-up duration and data availability varied among patients, and some patients were lost to follow-up. Accordingly, the post-30-day findings should be considered exploratory and descriptive and do not permit a definitive assessment of the long-term clinical effectiveness or prognosis associated with the single-hospitalization PCI–TAVR strategy. Fourth, the study lacked a contemporaneous comparator group of patients undergoing a conventional staged PCI and TAVR strategy across separate hospitalizations. In addition, the study population was heterogeneous with respect to both coronary anatomy and procedural strategy, including patients with single-vessel and multivessel CAD, as well as same-session PCI plus TAVR and staged PCI followed by TAVR during the same hospitalization. Although no significant association between procedural strategy and MACCE was observed in the present cohort, the relatively small number of staged cases limited the statistical power for subgroup analyses. Therefore, the relative safety and effectiveness of the single-hospitalization strategy compared with a conventional staged approach cannot be directly assessed, and the favorable outcomes reported here should be interpreted as descriptive rather than comparative. Finally, this study did not include detailed hemodynamic or imaging parameters such as total and procedure-specific contrast volume, fractional flow reserve, valve expansion symmetry, or prosthesis alignment angles, which may provide additional mechanistic insights into procedural outcomes and complications.

## 5. Conclusions

In conclusion, a single-hospitalization PCI and transfemoral TAVR strategy appeared feasible and was associated with acceptable short-term clinical outcomes in carefully selected patients. Reduced LVEF and mixed AS with AR were identified as independent predictors of MACCE. These findings inform clinical decision-making by highlighting the need for tailored risk stratification in patients with impaired ventricular function and complex valvular pathology. Given the absence of a contemporaneous comparator group, these findings should be interpreted as descriptive rather than comparative. Further large-scale, multicenter prospective studies are warranted to validate these results and optimize management strategies.

## Figures and Tables

**Table 1:** Demographic and clinical baseline characteristics of the study cohort by MACCE status.

Characteristics	Overall (*n* = 164)	MACCE (*n* = 28)	Non-MACCE (*n* = 136)	*p*-Value
Male, *n* (%)	95 (57.9%)	13 (46.4%)	82 (60.3%)	0.176
Age (years)	73.9 ± 7.2	73.7 ± 7.5	73.9 ± 7.1	0.902
BMI (kg/m^2^)	24.2 (22.6, 27.0)	25.1 (23.1, 27.7)	24.2 (22.5, 26.8)	0.375
NYHA ≥ III, *n* (%)	112 (68.3%)	24 (85.7%)	88 (64.7%)	0.030
EuroSCORE II (%)	4.8% (3.3%, 8.5%)	6.6% (4.7%, 11.7%)	4.4% (3.0%, 8.2%)	0.009
Comorbidities				
Hypertension, *n* (%)	114 (69.5%)	21 (75.0%)	93 (68.4%)	0.489
Diabetes, *n* (%)	52 (31.7%)	9 (32.1%)	43 (31.6%)	0.957
Myocardial infarction, *n* (%)	18 (11.0%)	4 (14.3%)	14 (10.3%)	0.515
Diseased coronary vessels, *n*	2.0 (1.0, 3.0)	2.0 (2.0, 3.0)	2.0 (1.0, 3.0)	0.811
Chronic kidney disease, *n* (%)	37 (22.6%)	6 (21.4%)	31 (22.8%)	0.875
Cerebrovascular disease, *n* (%)	16 (9.8%)	3 (10.7%)	13 (9.6%)	0.739
Previous cardiac intervention, *n* (%)	28 (17.1%)	5 (17.9%)	23 (16.9%)	1.000
Laboratory parameters				
Serum creatinine (µmol/L)	79.0 (66.8, 100.8)	93.0 (69.2, 112.6)	78.4 (66.2, 96.4)	0.098
Glucose (mmol/L)	6.0 (5.0, 7.5)	5.8 (4.8, 8.7)	6.0 (5.1, 7.4)	0.813
BNP (ng/L)	388.5 (148.2, 1041.2)	819.0 (347.0, 1532.2)	359.5 (139.2, 984.2)	0.050
Myoglobin (ng/mL)	29.0 (22.1, 46.8)	32.3 (24.5, 59.5)	28.2 (21.9, 42.6)	0.108
CK-MB (ng/mL)	2.2 (1.5, 3.4)	2.0 (1.5, 3.4)	2.2 (1.4, 3.2)	0.847
Troponin I (ng/L)	22.9 (9.2, 114.4)	44.2 (14.5, 392.1)	20.3 (8.8, 85.7)	0.080
Echocardiography				
Severe AS, *n* (%)	157 (95.7%)	26 (92.9%)	131 (96.3%)	0.342
Severe AR, *n* (%)	13 (7.9%)	5 (17.9%)	8 (5.9%)	0.049
Severe AS + AR, *n* (%)	6 (3.7%)	3 (10.7%)	3 (2.2%)	0.063
Severe MR, *n* (%)	24 (14.6%)	6 (21.4%)	18 (13.2%)	0.253
LVEF (%)	61.0 (54.0, 65.0)	55.0 (46.0, 58.0)	61.0 (55.2, 66.0)	<0.001
FS (%)	33.0 (30.0, 36.0)	29.0 (26.0, 31.2)	33.0 (30.0, 37.0)	<0.001
LVEDD (mm)	48.0 (44.0, 53.0)	51.0 (44.0, 56.5)	48.0 (43.2, 53.0)	0.317
LVESD (mm)	32.0 (28.0, 37.0)	33.5 (30.0, 43.8)	31.0 (27.2, 36.0)	0.086
Peak aortic jet velocity (cm/s)	452.5 (412.0, 501.2)	434.0 (397.8, 484.0)	454.5 (413.5, 502.5)	0.169
SPAP (mmHg)	31.0 (25.0, 42.0)	29.0 (24.5, 41.0)	32.0 (26.0, 43.0)	0.429

AR, aortic regurgitation; AS, aortic stenosis; BMI, body mass index; BNP, B-type natriuretic peptide; CK-MB, creatine kinase–MB isoenzyme; EuroSCORE II, European System for Cardiac Operative Risk Evaluation II; FS, fractional shortening; LVEDD, left ventricular end-diastolic diameter; LVESD, left ventricular end-systolic diameter; LVEF, left ventricular ejection fraction; MACCE, major adverse cardiovascular and cerebrovascular events; MR, mitral regurgitation; NYHA, New York Heart Association functional class; SPAP, systolic pulmonary artery pressure. Note: Given the small number of MACCE, the between-group comparisons in this table are exploratory and may be underpowered. Borderline *p* values should be interpreted cautiously.

**Table 2:** Procedural characteristics, early clinical outcomes, and individual components of MACCE according to MACCE status.

Characteristics	Overall (*n* = 164)	MACCE (*n* = 28)	Non-MACCE (*n* = 136)	*p*-Value
Procedural characteristics and outcomes				
PCI and TAVR on the same day, *n* (%)	130 (79.3%)	25 (89.3%)	105 (77.2%)	0.203
Number of implanted stents, *n*	1.0 (1.0, 2.0)	1.0 (1.0, 2.0)	1.0 (1.0, 2.0)	0.520
PCI-related procedural complication, *n* (%)	0 (0%)	0 (0%)	0 (0%)	-
Conversion to open surgery, *n* (%)	0 (0%)	0 (0%)	0 (0%)	-
Device success, *n* (%)	160 (97.6%)	26 (92.9%)	134 (98.5%)	0.135
Post-procedural complications				
Acute kidney injury, *n* (%)	18 (11.0%)	9 (32.1%)	9 (6.6%)	<0.001
Mechanical circulatory support, *n* (%)	4 (2.4%)	4 (14.3%)	0 (0%)	<0.001
Blood transfusion, *n* (%)	19 (11.6%)	9 (32.1%)	10 (7.4%)	0.001
Gastrointestinal bleeding, *n* (%)	4 (2.4%)	4 (14.3%)	0 (0%)	<0.001
Deep vein thrombosis, *n* (%)	2 (1.2%)	2 (7.1%)	0 (0%)	0.028
Sepsis, *n* (%)	2 (1.2%)	0 (0%)	2 (1.5%)	1.000
Permanent pacemaker implantation, *n* (%)	2 (1.2%)	0 (0%)	2 (1.5%)	1.000
Post-procedural echocardiographic outcomes				
LVEF, %	60.0 (54.0, 65.0)	58.0 (50.5, 62.0)	60.0 (55.2, 65.0)	0.060
Peak aortic jet velocity, cm/s	229.0 (186.8, 264.8)	233.5 (187.2, 266.0)	228.0 (187.0, 259.0)	0.766
Individual components of MACCE				
In-hospital death, *n* (%)	6 (3.7%)	6 (21.4%)	-	-
Stroke, *n* (%)	6 (3.7%)	6 (21.4%)	-	-
Myocardial infarction, *n* (%)	1 (0.6%)	1 (3.6%)	-	-
Major bleeding, *n* (%)	4 (2.4%)	4 (14.3%)	-	-
Cardiogenic shock, *n* (%)	6 (3.7%)	6 (21.4%)	-	-
Cardiac arrest, *n* (%)	4 (2.4%)	4 (14.3%)	-	-
Repeat revascularization, *n* (%)	0 (0%)	0 (0%)	-	-
Rehospitalization for heart failure, *n* (%)	8 (4.9%)	8 (28.6%)	-	-

LVEF, left ventricular ejection fraction; MACCE, major adverse cardiovascular and cerebrovascular events; PCI, percutaneous coronary intervention; TAVR, transcatheter aortic valve replacement. Individual MACCE components were not mutually exclusive; therefore, their summed frequencies may exceed the total number of patients with MACCE.

**Table 3:** Factors associated with MACCE identified by univariable and multivariable logistic regression.

Variables	Univariable OR (95% CI)	*p*-Value	Multivariable OR (95% CI)	*p*-Value
Pre-procedural LVEF (per 1% increase)	0.955 (0.924–0.988)	0.008	0.955 (0.920–0.992)	0.017
Mixed severe AS with AR (yes vs. no)	5.320 (1.015–27.878)	0.048	6.363 (1.112–36.405)	0.038

MACCE, major adverse cardiovascular and cerebrovascular events; LVEF, left ventricular ejection fraction; AS, aortic stenosis; AR, aortic regurgitation.

## Data Availability

The data that support the findings of this study are available from the corresponding author upon reasonable request.

## Abbreviations

The following abbreviations are used in this manuscript:

AKI	Acute kidney injury
AR	Aortic regurgitation
AS	Aortic stenosis
AVD	Aortic valve disease
BMI	Body mass index
BNP	B-type natriuretic peptide
CAD	Coronary artery disease
CI	Confidence interval
CK-MB	Creatine kinase–MB isoenzyme
ECMO	Extracorporeal membrane oxygenation
FS	Fractional shortening
IABP	Intra-aortic balloon pump
IQR	Interquartile range
LM	Left main
LVESD	Left ventricular end-systolic diameter
LV	Left ventricle
LVEDD	Left ventricular end-diastolic diameter
LVEF	Left ventricular ejection fraction
MACCE	Major adverse cardiovascular and cerebrovascular events
MAVD	Mixed aortic valve disease
MR	Mitral regurgitation
NYHA	New York Heart Association
OR	Odds ratio
PCI	Percutaneous coronary intervention
PPI	Permanent pacemaker implantation
PVL	Paravalvular leak
SPAP	Systolic pulmonary artery pressure
SYNTAX	Synergy between PCI with Taxus and Cardiac Surgery
TAVR	Transcatheter aortic valve replacement
TIMI	Thrombolysis in Myocardial Infarction
VARC	Valve Academic Research Consortium
